# Novel Agents in Chronic Lymphocytic Leukemia: New Combination Therapies and Strategies to Overcome Resistance

**DOI:** 10.3390/cancers13061336

**Published:** 2021-03-16

**Authors:** Moritz Fürstenau, Barbara Eichhorst

**Affiliations:** 1 German CLL Study Group, Center for Integrated Oncology Aachen Bonn Cologne Duesseldorf (CIO ABCD), Department I of Internal Medicine, University Hospital Cologne, University of Cologne, 50937 Cologne, Germany; moritz.fuerstenau@uk-koeln.de; 2 Cancer Center Cologne Essen (CCCE)—Partner Site Cologne, University of Cologne, 50937 Cologne, Germany

**Keywords:** chronic lymphocytic leukemia, drug resistance, novel agents, combination treatment

## Abstract

**Simple Summary:**

Nowadays, many patients with chronic lymphocytic leukemia (CLL) are treated with so-called novel agents, including BTK inhibitors, Bcl-2 inhibitors and PI3K inhibitors. As CLL is a chronic disease, most patients will relapse on or after treatment with these drugs and various mechanisms behind this resistance to novel agents have been described. In this review, we present the current evidence on resistance to novel agents, discuss approaches to prevent its development and provide guidance on the treatment of patients who have already acquired resistance.

**Abstract:**

The approval of Bruton’s tyrosine kinase (BTK) inhibitors such as ibrutinib and acalabrutinib and the Bcl-2 inhibitor venetoclax have revolutionized the treatment of chronic lymphocytic leukemia (CLL). While these novel agents alone or in combination induce long lasting and deep remissions in most patients with CLL, their use may be associated with the development of clinical resistance. In this review, we elucidate the genetic basis of acquired resistance to BTK and Bcl-2 inhibition and present evidence on resistance mechanisms that are not linked to single genomic alterations affecting these target proteins. Strategies to prevent resistance to novel agents are discussed in this review with a special focus on new combination therapies.

## 1. Introduction

Over the last decade, the increasing knowledge on the pathogenesis and disease-driving mechanisms of chronic lymphocytic leukemia (CLL) has finally translated into a multitude of new treatment options for patients with CLL [1,2]. The approvals of the Bruton’s tyrosine kinase (BTK) inhibitors ibrutinib and more recently acalabrutinib as well as the B-cell lymphoma 2 (Bcl-2) antagonist venetoclax have transformed the treatment paradigm in both treatment-naïve and relapsed/refractory CLL [3,4,5,6,7,8,9,10,11,12]. While venetoclax is mostly used in combination with anti-CD20 antibodies for a fixed duration of one (firstline) to two years (relapse setting), BTK inhibitors (BTKi) and PI3K inhibitors are exclusively approved for continuous treatment and mainly prescribed as monotherapies.

Despite these new therapeutic options, CLL remains a chronic, incurable disease, with allogeneic hematopoietic stem cell transplantation being the only treatment with confirmed curative potential in a minority of patients [13,14,15,16,17]. Patients treated successfully with kinase inhibitors or venetoclax eventually relapse/progress or have to stop treatment due to intolerance. The mechanisms behind these clinical manifestations of resistance have been studied, and are in part explained by genetic resistance mechanisms [18,19,20]. Besides mutations in the drug-targeting proteins, several non-genetic mechanisms rendering CLL cells resistant to treatment have been described [21,22,23,24]. In this article, we will review the current state of research on resistance to novel agents, propose strategies to avoid resistance and provide guidance on treating patients who relapse or progress on novel agents.

## 2. Mechanisms of Resistance to BTK Inhibition

After proving exceptional activity in all analyzed patient groups, ibrutinib was approved for the treatment of CLL by the U.S. Food and Drug Administration (FDA) as well as the European Medicines Agency (EMA) in the relapsed/refractory setting in 2014 and for frontline use in 2016 [3,4,5,9,12]. The second-generation BTKi acalabrutinib was recently approved based on marked survival benefits in two randomized controlled trials in treatment-naïve and relapsed/refractory CLL [10,25]. In the US a third BTKi, zanubrutinib, has been approved for the treatment of mantle cell lymphoma and is expected to be approved for CLL soon [26].

While more recently, ibrutinib and acalabrutinib have been tested within fixed-duration combinations, these combinations of novel agents are not yet approved. Hence, their main use remains as monotherapy that is given until disease progression or unacceptable toxicity [27,28,29,30]. Adding to the financial burden and side effects associated with an indefinite therapy, the continued exposure to BTKi seems to promote the acquisition of resistance mutations.

### 2.1. Genetic Mechanisms of Resistance to BTK Inhibition

Ibrutinib, acalabrutinib and other covalent binding BTK inhibitors exhibit their main inhibitory effect on the B-cell receptor (BCR) pathway by irreversibly binding to the C481 position of BTK and thereby inactivating the enzyme. Not surprisingly, the first discovered mutations that conferred resistance to ibrutinib were mutations in the *BTK* gene at the ibrutinib binding site [11]. Woyach and colleagues performed whole exome sequencing on six patients progressing on ibrutinib and discovered that five of these patients had acquired cysteine-to-serine mutations in *BTK* at position 481 (C481S) that were not present before treatment with ibrutinib. Through the replacement of cysteine with serine, ibrutinib can no longer bind to BTK irreversibly, leading to restored BCR signaling and clinical resistance to ibrutinib [11]. Sequencing data from early trials have shown *BTK* mutations in approximately 80% of patients progressing on ibrutinib [11,31,32,33]. By sequencing earlier samples of those progressing patients, *BTK*-mutated clones were already detected at a median of eight and nine months before clinical progression [31,33]. In a large cohort comprising 373 patients treated with ibrutinib at the Ohio State University (OSU), BTK mutations occurred in 23.3% at a median of 34 months following ibrutinib initiation [32]. More recently, an analysis of a French registry cohort has shown an incidence of BTK mutations of 57% in patients who were still on ibrutinib, suggesting that a substantial proportion of patients with CLL on ibrutinib monotherapy already harbors resistance-conferring mutations when clinically still responding to treatment [34]. Importantly, the acquisition of *BTK* mutations was clearly associated with the risk of subsequent clinical progression in this study, confirming findings of Woyach et al. who had also studied this phenomenon prospectively [33,34].

On rare occasions, other putatively resistance-conferring mutations have been reported in *BTK*. Some also affect the ibrutinib binding site (e.g., C481F, C481Y) [32], while some others affect BTK binding to B-cell linker protein (BLNK), enabling Phospholipase C gamma 2 (PLCy2) activation despite the presence of ibrutinib [35].

*BTK* mutations were often found to be accompanied by mutations in *PLCG2*, the gene coding for PLCy2, the substrate downstream of BTK [11,31,32,33,34]. Most *PLCG2* mutations are gain-of-function mutations at the SH2/SH3 domain of the *PLCG2* gene, leading to autonomous BCR signaling despite BTK inhibition [32]. In two prospective studies, the cumulative incidence of *PLCG2* mutations was 13% after at least 3 years of ibrutinib treatment and 10% after a median of 3 years after start of ibrutinib, respectively [32,34]. The majority of patients with *PLCG2* mutations harbored concurrent *BTK* mutations that occurred at similar time points in treatment (median 35 and 34 months after start of ibrutinib, respectively). In the context of extensive sequencing efforts, other rare genetic aberrations possibly conferring resistance to ibrutinib have been described, including del(8p) and 2p gain with subsequent *XPO1* overexpression [36,37].

A recent study assessed the occurrence of *BTK* and *PLCG2* mutations in a pooled cohort of 388 patients without clinical progression from various ibrutinib trials by next-generation sequencing [38]. With a median follow-up on treatment of 35 months (previously untreated patients) and 36 months (relapsed/refractory patients), the analysis revealed that the incidence of these resistance-conferring mutations strongly differed between previously untreated patients and patients receiving ibrutinib in a relapsed/refractory setting. Among patients on first-line ibrutinib, only 3% harbored *BTK* mutations while 30% of the relapsed/refractory patients showed *BTK* mutations, consistent with differences in clinical progression rates between these populations.

The pattern of genetically mediated resistance to acalabrutinib or other covalent binding BTKis such as zanubrutinib is thought to be similar to ibrutinib due to their formation of a covalent bond at the same binding site. The only study on resistance mutations in CLL patients treated with acalabrutinib in the frontline as well as relapse setting detected *BTK* C481 mutations in 69% of patients progressing on acalabrutinib, while 14% of these patients harbored concurrent subclonal *PLCG2* mutations [39].

In patients with progressing CLL, *BTK* and *PLCG2* mutations are often found in clones/subclones of variable size with reported ranges between 0.2% and nearly 100% [11,31,32,33,34]. However, even small subclones might lead to clinical resistance, as studies in Waldenström’s macroglobulinemia (WM) and diffuse large B-cell lymphoma (DLBCL) suggest. In vitro and in vivo assays in those entities showed that *BTK*-mutant cells protect wildtype cells from ibrutinib-induced killing by releasing interleukin 6 (IL-6) and IL-10 and thereby triggering strong prosurvival signaling including the JAK/STAT pathway, providing a possible explanation for clinical resistance in patients with a *BTK*-mutated subclone [40]. Furthermore, the variant allele frequency (VAF) of *BTK* mutations in circulating CLL cells appeared to be lower in patients with primarily nodal relapses indicating that genetic aberrations detected in peripheral blood CLL cells are not necessarily representative for clonal composition in other compartments [33].

### 2.2. Non-Genetic Mechanisms of Resistance to Ibrutinib

Despite comprehensive genetic analyses, a substantial part of clinical progressions on ibrutinib are not explained by genetic alterations. Different non-genetic mechanisms of adaptation to ibrutinib treatment have been described in CLL cells. The main mechanisms are the maintenance of BCR signaling through alternative pathways and interactions of CLL cells and the tumor microenvironment (TME).

Under the influence of BTK inhibition by targeted drugs, CLL cells and malignant B-cells in other lymphoid neoplasms may adapt and compensate for the blocked BTK axis by activating the PI3K/Akt/Erk pathway [21,22,41]. Functional analyses by Spina et al. in cells from ibrutinib-treated patients revealed that the BCR pathway through Akt and Erk was still inducible upon stimulation of the B-cell receptor in spite of effective inhibition of the BTK/PLCy2 pathway [22]. The group also showed that in CLL cells persisting under ibrutinib, genes involved in the MAPK/Erk pathway were upregulated [21]. Similarly, CD40L stimulation of the non-canonical NF-kB pathway still led to nuclear translocation of NF-kB while the canonical NF-kB pathway was inhibited by ibrutinib [22].

Ibrutinib treatment was shown to effectively reduce chemokines involved in homing, retention and adhesion of CLL cells in their growth- and survival-supporting microenvironment [42,43]. During BTK inhibition CLL cells may adapt their phenotype by upregulation of homing/adhesion proteins and increased surface IgM [22,44,45]. Protective nurse-like cells (NLC) in the TME also seem to play a role in rescuing CLL cells from ibrutinib-induced killing, as ibrutinib does neither seem to fully antagonize the CLL cell-supporting function of NLCs nor to effectively mobilize them from their lymph node or bone marrow niches [46,47,48]. It has also been hypothesized that extracellular vesicles of bone marrow stromal cells play a role in the development of drug resistance by rescuing CLL cells from apoptosis and thereby increasing chemoresistance to different drugs, including ibrutinib, idelalisib, venetoclax and fludarabine [49].

## 3. Mechanisms of Resistance to PI3K Inhibitors

Two inhibitors of PI3K, idelalisib and duvelisib, have been approved for the treatment of relapsed/refractory CLL. Both drugs are used as continuous therapies and mostly applied in heavily pretreated patients, hence progression on these agents was observed comparably early with a median PFS of 16.4 months for idelalisib plus rituximab and 13.3 months for duvelisib in the trials leading to approval [50,51]. In spite of various sequencing efforts and in contrast to BTKi and venetoclax, no resistance-conferring mutations were so far identified in the gene coding the target protein PI3K [52,53]. Several mechanisms like activating mutations in MAPK pathway genes, an upregulation of *Igf1r* and an amplification or activating mutations of *PI3KCA* have been associated with resistance to idelalisib [53,54,55,56]. However, in the absence of comprehensive analyses, it remains largely unclear whether these mechanisms are responsible for clinical resistance in a substantial proportion of patients.

## 4. Mechanisms of Venetoclax Resistance

B-cell lymphoma-2 (Bcl-2) is an anti-apoptotic protein and part of a family of proteins, regulating B cells disposition to undergo apoptosis. In CLL, overexpression of Bcl-2 results in an inhibition of pro-apoptotic BH3-only proteins ensuring survival of the CLL cell [57,58,59]. Venetoclax, a BH3 mimetic, has been developed to bind Bcl-2 at the same site as BH3-only proteins to effectively inhibit Bcl-2 [60]. After clinical studies in CLL have consistently shown impressive activity of venetoclax in all therapeutic settings as monotherapy as well as in combinations, it has been approved for the treatment of patients with previously untreated and relapsed/refractory CLL [7,8,27,28,29,30,61,62,63,64,65,66].

### 4.1. Mutations in BCL2 and Alterations in Cancer-Related Genes

The first discovered resistance-conferring mutation in the context of venetoclax treatment affects the binding site of the target protein, Bcl-2. In a study of 15 patients progressing on venetoclax, Blombery and colleagues could identify the *BCL2* single-nucleotide variant G101V in seven of 15 patients by next-generation sequencing (NGS) and digital-droplet polymerase chain reaction (ddPCR) [20]. The variant was not detected in these patients before initiation of venetoclax treatment and in a large group of venetoclax-unexposed patients with CLL. Using earlier samples of the seven patients, the mutation could already be found up to 25 months before clinical relapse [20]. In a functional analysis, Blombery et al. could demonstrate that the ability of venetoclax to compete with BH3-only proteins for binding of Bcl-2 was strongly impaired in the presence of the G101V mutation [20]. Structural analyses revealed the molecular basis of this reduction in affinity by reporting the crystal structure of Bcl-2 in complex with venetoclax [67].

Recently, a more sensitive sequencing approach revealed the presence of numerous other *BCL2* mutations in patients with G101V variants who clinically progressed on venetoclax monotherapy administered as relapse treatment [68]. Ten out of 11 (91%) of the analyzed patients harbored additional acquired *BCL2* mutations in different CLL cells with strongly varying cancer cell fractions [68]. Another report identified a putatively resistance-conferring D103Y mutation in *BCL2*, also affecting the binding site of venetoclax [18].

Given the often subclonal nature of the identified mutations in clinically progressing patients and the lack of *BCL2* mutations in a large proportion of patients who relapse on venetoclax, it is questionable if *BCL2* mutations are the sole cause of clinical resistance to venetoclax [69,70].

Another study of eight high-risk CLL patients carrying del(17p) and/or *TP53* mutations progressing on venetoclax revealed other recurrent aberrations with resistance-conferring potential and described a rather heterogeneous clonal evolution in venetoclax-treated CLL [19]. Whole exome sequencing in this cohort identified acquired homozygous *CDKN2A/B* deletion in 3/8 (38%) and *BTG1* missense mutations in 2/8 (25%) patients. Single patients harbored mutations in *BRAF*, *SF3B1*, *RB1*, *MLL3*, *BIRC3* and a high-level focal amplification of CD274 (PD-L1) [19]. While further functional analyses showed no increased resistance in *CDKN2A/B*-mutated cells, the *BRAF* mutation was associated with elevated Mcl-1 expression and showed resistance in transduced cell lines.

### 4.2. Changes in Cellular Metabolism/Bcl-2 Family Members

Resistance to venetoclax treatment has also been detected on a non-mutational basis through upregulation of other anti-apoptotic Bcl-2 family members. As the sensitivity to BH3 mimetics depends largely on the ratio of the expression of proapoptotic and antiapoptotic proteins, it was hypothesized early on that an increase in anti-apoptotic proteins following venetoclax exposure would confer resistance to Bcl-2 inhibition [71,72]. Various studies have demonstrated that venetoclax-associated overexpression of Mcl-1 and Bcl-X_L_ can confer resistance to the drug in vitro and in vivo [23,24,72,73,74,75,76]. Recently it has been postulated that among the Bcl-2 family members, Bcl-X_L_ is more relevant for the development of venetoclax resistance, as functional analyses have shown that proapoptotic proteins preferably interact with Bcl-X_L_ when both anti-apoptotic proteins are present [24]. Guièze and colleagues have performed an extensive analysis including genome-scale screens in a Bcl-2-driven lymphoma cell line and integrated expression profiling and identified Mcl-1 overexpression and BIM sequestration as a resistance-conferring mechanism to venetoclax treatment [23]. The group has also shown that reprogramming of the biology of the mitochondrial outer membrane can result in altered expression of Bcl-2 family members and an increase in oxidative phosphorylation (OXPHOS) activity, with both leading to resistance to Bcl-2 inhibition [23].

**Figure 1:**
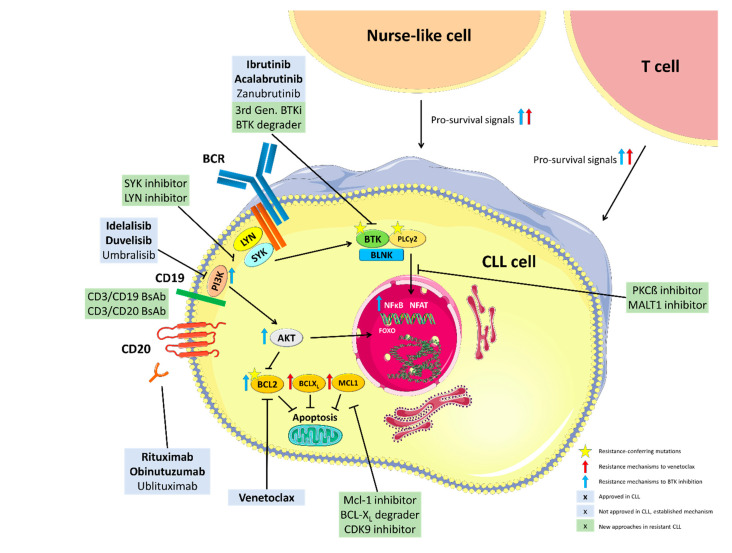
Mechanisms of resistance to BTK and Bcl-2 inhibition and targets of approved and experimental treatment approaches. BCR: B cell receptor, PI3K: Phosphoinositide 3-kinase, BTK: Bruton’s Tyrosine Kinase, PLCy2: Phospholipase gamma 2, BLNK: B cell linker protein, PKCß: Protein kinase C beta, MALT1: Mucosa-associated lymphoid tissue lymphoma translocation protein 1, Mcl-1: myeloid cell leukemia 1, BCL-XL: B-cell lymphoma-extra large, CDK9: Cyclin-dependent kinase 9, Bcl-2: B-cell lymphoma 2, NFkB: Nuclear factor kappa B, FOXO: Forkhead transcription factors, NFAT: Nuclear factor of activated T-cells, BsAb: Bispecific Antibody. This figure was produced by Moritz Fürstenau using servier medical art (smart.servier.com, accessed on 1 February 2021).

The above outlined mechanisms of resistance and potential approaches to overcome them are summarized in Figure 1.

## 5. Strategies to Prevent Resistance to Novel Agents

The above cited studies have demonstrated that resistance to novel agents in CLL, especially when caused by acquired *BTK* and *BCL2* mutations, appears to occur rather late in the course of treatment, though some reports show the detection of these mutations as early events [31]. A recent analysis by Wiestner and colleagues showed that resistance mutations in *BTK* or *PLCG2* seem to appear later in patients receiving ibrutinib as first-line therapy in comparison to patients in a relapsed/refractory situation [38]. There are various promising strategies to possibly circumvent resistance to BTKi and Bcl-2 inhibitors.

1. In the pivotal studies, acquired *BTK* and *BCL2* mutations could be detected months and even years [11,21] before the patients fulfilled the iwCLL criteria [77] of clinical disease progression. Following these observations, Woyach and colleagues initiated a prospective study on 112 patients receiving ibrutinib monotherapy and performed serial screening for known resistance mutations [33]. They demonstrated that mutations in *BTK* and *PLCG2* occurred early and clearly correlated with consequent clinical disease progression and could thus be used as a biomarker for relapse and an opportunity to adapt treatment [33]. Hence, a future strategy could be to monitor resistance similarly to strategies in antiviral treatments as for example HIV.
2. Constant selection pressure by administering continuous monotherapies with BTKis or Bcl-2 inhibitors might contribute to the acquisition of resistance mutations in a significant number of patients. Hence, avoiding constant drug exposure and selection of BTKi- and venetoclax-resistant clones by using time-limited treatment approaches could be another option to circumvent the development of resistance.
3. Another promising strategy to circumvent the acquisition of resistance to novel agents is the development of next generation inhibitors which bind non-covalently to the target kinase and are therefore still active in CLL cells harboring the most common resistance mutations.

The strategies of time-limited combination as well as the current status of the development of non-covalent BTK inhibitors is discussed in the following segment.

### 5.1. Time-Limited Combination Treatments

Time-limited approaches include combinations of different drugs, as monotherapies usually do not achieve sufficiently deep responses that would allow drug discontinuation [78,79]. Combination therapies would possibly also reduce the selection of *BTK*- or *BCL2*-mutated clones, as e.g., *BTK* C481S-mutated cells could still be eliminated by concomitant venetoclax or an anti-CD20 antibody while they would likely outgrow under ibrutinib monotherapy. On the other hand, it has been shown that the combination of venetoclax with a BTKi was able to reprogram apoptotic dependencies and venetoclax resistance could be overcome in malignant B cells [24,80,81]. Also, resistance-mediating upregulation of Bcl-2 under ibrutinib monotherapy can increase the sensitivity towards venetoclax [82,83]. Another probable advantage of combination therapies would be the lower minimal residual disease (MRD) these combination treatments could achieve. A CLL cell count of <10^−6^ which was shown to be achieved by a substantial fraction of patients receiving a time-limited combination of venetoclax and obinutuzumab correlates to 10,000 times less measurable CLL burden compared to a MRD-positive (≥10^−2^) patient under ibrutinib monotherapy [84]. It is conceivable that in a substantially smaller CLL cell pool, resistance-conferring genetic alterations are less likely to develop. In addition, avoiding resistance by limiting duration and combining different drugs would possibly allow for re-exposure to the same drug combination. Data from studies evaluating the most promising time-limited combination treatments is listed in Table 1, while currently ongoing trials are shown in Table 2.

**Table 1:** Selected results of studies on time-limited combination strategies.

Name/Identifier	Experimental Treatment Arm	Phase	TN vs. R/R	Efficacy (Experimental Treatment Arm)	Reference
Venetoclax + anti-CD20 antibody					
MURANO NCT02005471	Venetoclax + rituximab	3	R/R	Post-treatment uMRD rate: 62% 5-year PFS/ 5-year OS: 51.1%/82.1%	Seymour et al. 2018 [8]
CLL14 NCT02242942	Venetoclax + obinutuzumab	3	TN	Post-treatment uMRD: 75.5% 4-year PFS/ 4-year OS: 74%/85.3%	Fischer et al. 2019 [7]
CLL2-BAG NCT02401503	(Bendamustine) + venetoclax + Obinutuzumab	2	TN, R/R	Post-treatment uMRD: 87% 15-month PFS/ 15-month OS: 92%/95%	Cramer et al. 2018 [63]
Venetoclax + BTK inhibitor					
CAPTIVATE NCT02910583	Venetoclax + ibrutinib	2	TN	1-year uMRD rate: 73% 30-month PFS: >95%	Wierda et al. 2020 [85]
NCT02756897	Venetoclax + ibrutinib	2	TN	1-year uMRD rate: 61% 1-year PFS/1-yeary OS: 98%/99%	Jain et al. 2019 [28]
CLARITY 2015-003422-14	Venetoclax + ibrutinib	2	TN	1-year uMRD rate: 53%	Hillmen et al. 2019 [27]
VISION NCT03226301	Venetoclax + ibrutinib	2	R/R	15-month uMRD rate: 55%	Niemann et al. 2020 [29]
Triple combinations					
NCT02427451	Venetoclax + ibrutinib + obinutuzumab	2	TN, R/R	Post-treatment uMRD rate TN: 67% Post-treatment uMRD rate R/R: 50%	Rogers et al. 2020 [30]
CLL2-GIVe NCT02758665	Venetoclax + ibrutinib + obinutuzumab	2	TN	Post-treatment uMRD rate: 81%	Huber et al. 2020 [86]
CLL-003 NCT02296918	Acalabrutinib + venetoclax + obinutuzumab	1b	TN, R/R	10-month uMRD rate: 71% 18-month PFS/18-month OS: 100%/100%	Woyach et al. 2020 [87]
NCT03580928	Acalabrutinib + venetoclax + obinutuzumab	2	TN	16-month uMRD rate: 84%	Davids et al. 2020 [88]
NCT03824483	Zanubrutinib + venetoclax + obinutuzumab	2	TN	Overall uMRD rate: 92%	Soumerai et al. 2020 [89]

TN: treatment-naïve, R/R: relapsed/refractory, uMRD: undetectable minimal residual disease (<10^−4^), PFS: progression-free survival, OS: overall survival.

Two time-limited combinations have recently been approved for the treatment of CLL. After the MURANO study demonstrated significantly superior survival in relapsed/refractory patients treated with 24 months of venetoclax and rituximab compared to bendamustine and rituximab, the scheme was approved and widely adopted in the relapsed/refractory setting [8]. Follow-up publications showed durable responses in patients treated with venetoclax and rituximab with 57% of the patients still being progression-free 4 years after the start of treatment [90]. No information on the occurrence of *BCL2* mutations has been published so far, but first data of patients being retreated with the same combination at progression suggest that a large part of patients can respond to a re-exposure after time-limited venetoclax and rituximab [91].

More recently, the one-year combination treatment of venetoclax and obinutuzumab was approved after yielding impressive PFS advantages and high rates of undetectable MRD (uMRD) when compared to chlorambucil and obinutuzumab [7,61]. The latest update showed durable and deep responses and it could even be demonstrated that the rate with which MRD increases after the end of treatment is lower in the venetoclax and obinutuzumab arm, suggesting that not only the depth but also the quality of the response is improved [84]. As only a few patients have progressed after this combination, retreatment data is scarce, but it is conceivable that patients might respond again to venetoclax and obinutuzumab, as they have only been exposed for one year. Clinical trials evaluating retreatment after time-limited combinations are currently being planned.

There are currently numerous studies ongoing testing combination approaches in all stages of clinical development [92]. The most promising combination therapies consist of a BTKi in combination with venetoclax with or without the anti-CD20 antibody obinutuzumab and are currently studied in phase 3 trials.

Jain and colleagues published an early interim analysis of their phase 2 trial of a fixed-duration treatment with ibrutinib and venetoclax in treatment-naïve patients with CLL [28]. Ibrutinib was given alone for 3 cycles (28 days each) followed by 24 cycles of combined treatment. After one year of combination treatment, 88% of the evaluable patients had a complete remission or complete remission with incomplete count recovery and 61% of the evaluable patients showed uMRD (<10^−4^). However, due to the early read-out, longer observation is required. An overview on selected other studies is shown in Table 1. Results from the Phase 3 FLAIR (2013-001944-76) and GLOW (NCT03462719) trials, testing this combination in a randomized setting, are eagerly awaited and expected soon. The phase 3 CLL17 trial of the German CLL Study Group (GCLLSG) (NCT04608318) is currently evaluating the approved options ibrutinib monotherapy and venetoclax plus obinutuzumab against ibrutinib and venetoclax.

The shorter triple combination of ibrutinib, venetoclax and obinutuzumab was studied extensively as well. A phase 2 study of the combination in treatment-naïve and relapsed/refractory patients with CLL was recently published and yielded high response rates, high rates of uMRD and durable remissions [30]. The same combination was studied in the phase 2 CLL2-GIVe trial, in which patients with detectable post-treatment MRD could continue ibrutinib treatment until cycle 36. After 15 months of combined treatment, 33 of 41 patients (81%) achieved uMRD in the peripheral blood and 22 patients stopped treatment at this time point due to uMRD and CR/CRi [86]. The same concept was tested in the randomized phase 3 GAIA/CLL13 trial (NCT02950051) by the GCLLSG against standard chemoimmunotherapy and other fixed-duration venetoclax combinations. The study will also be the first to show results on the efficacy of the time-limited combinations of venetoclax plus obinutuzumab in fit patients as well as venetoclax plus rituximab in the first-line setting.

Similar combination treatments using acalabrutinib instead of ibrutinib have yielded similarly promising results in phase 2 trials and are currently being evaluated against standard chemoimmunotherapy in a large phase 3 trial (NCT03836261) [87,88].

The GCLLSG currently runs two trials evaluating time-limited, MRD-driven combination treatments of venetoclax, obinutuzumab and acalabrutinib/zanubrutinib after an optional debulking with bendamustine (NCT03787264, NCT04515238). The trials are accompanied by an extensive real-time screening program for the occurrence of known resistance mutations to BTKi and venetoclax as well as other potentially druggable targets using ddPCR on circulating tumor DNA (ctDNA). In the induction phase, the screening is performed monthly followed by every three months and in the case of occurring resistance mutations, the individual results are discussed by the sponsor and the treating physician. The concept is currently too labor-intensive and costly to apply it in clinical practice, but it will certainly provide valuable information on the relevance of known resistance mutations and their early detection in time-limited combination approaches.

### 5.2. Non-Covalent BTK Inhibitors and BTK Degraders

Second-generation BTK inhibitors like acalabrutinib and zanubrutinib have demonstrated their efficacy and safety in different B-cell malignancies and might be considered as an alternative to ibrutinib due to fewer off-target effects and different safety profiles [6,10,25,93,94]. However, they also bind irreversibly to BTK at the same binding site as ibrutinib and are also not able to inhibit BTK in patients with the most common mutation associated with ibrutinib resistance (*BTK* C481S).

Novel, third-generation, non-covalent BTK inhibitors have been designed to overcome this mechanism of resistance by reversibly binding to BTK without interacting with C481. Two of the new third-generation BTKi, vecabrutinib and fenebrutinib, have shown initial proof of concept but do not seem to be further evaluated in CLL [95,96]. Two other third-generation BTKi, ARQ 531 and LOXO-305, have yielded promising results in their early clinical trials and were advanced to the next stage of clinical testing [97,98]. ARQ 531 is a non-covalent BTKi that binds to the ATP-binding region of BTK and has shown its activity in a phase 1 study in relapsed/refractory CLL patients [98]. In a heavily pretreated population with 22/26 (85%) patients harboring *BTK* C481S mutations, seven patients achieved a partial response (PR). The phase 2 part of the study is currently ongoing. LOXO-305 is a non-covalent BTKi that has demonstrated its efficacy in a phase 1/2 trial of patients with relapsed/refractory CLL [97]. In the BRUIN study, of 65 patients with CLL/SLL (58 BTKi-pretreated, 7 previously BTKi-naïve) who had a response assessment, 37 (57%) either had a PR or PR with lymphocytosis and only one patient progressed so far. The median follow-up of the study is still short and responses are thought to improve over time, as seen in other BTK inhibitors.

Proteolysis targeting chimera (PROTAC) have been developed to target proteins that are otherwise difficult to inhibit. They consist of a ligand directed against the target of interest coupled with a ligand for binding of an E3 ubiquitin ligase and act by facilitating degradation of the target by the proteasome [99]. Multiple BTK-targeting PROTACs were developed and the first analysis specifically examining their activity in *BTK* C481S-mutated CLL demonstrated activity of the construct MT-802, whereas ibrutinib expectedly showed no relevant activity in mutant cells [100]. Another BTK-targeted PROTAC, L18I, was able to induce degradation of *BTK* C481S-mutated cells as well as other BTK variants with Thr, Gly, Trp or Ala substitutions at Cys [101]. Compared with ibrutinib, PROTACs also seem to more selectively target BTK, whether this translates to less clinical toxicity will however only be clear when PROTACs are tested in clinical trials. PROTACs against Bcl-X_L_ have also been reported and could be promising drugs for patients resistant to BTKi and venetoclax [102]. Due to its Von Hippel-Lindau E3 ligase it is thought to not relevantly degrade Bcl-X_L_ in platelets, which might lead to less thrombocytopenia when compared to previous Bcl-X_L_ inhibitors like ABT263 and might therefore be more tolerable [102].

## 6. Treatment Options in Case of Clinical Resistance to Novel Agents

Time-limited combination treatments might effectively prevent the acquisition of certain types of resistance, but currently indefinite monotherapies are widely used in the frontline setting and it is important to know how to best treat patients with acquired resistance to those drugs. The next section will focus on experimental treatment options for patients relapsing or progressing on approved novel agents but begin with a short overview of the current evidence on optimal sequencing of approved treatment options. Approved and experimental cellular treatment options like allogeneic hematopoietic stem cell transplant (allo HCT), Chimeric Antigen Receptor (CAR) T-cell and CAR NK-cell therapies will not be discussed in this review.

### 6.1. Optimal Sequencing of Approved Agents

Some patients may be resistant to either BTKi or venetoclax but have not yet been exposed to the other and studies have recently demonstrated that both, venetoclax after ibrutinib and BTKi after venetoclax are effective treatment options for patients who have discontinued treatment with either of the drugs [103,104]. In earlier retrospective analyses by Mato and colleagues, idelalisib appeared inferior to both, ibrutinib after venetoclax and venetoclax after ibrutinib [105]. Given the overall superior efficacy data of venetoclax and BTKi compared to PI3K inhibitors in CLL, a sequencing approach using venetoclax-based regimens after progression on ibrutinib and BTKi after progression on venetoclax is the current standard of care [92,106]. While patients discontinuing ibrutinib due to toxicities can be treated with acalabrutinib as the efficacy and safety of this approach has recently been demonstrated, this is not the case for patients who progress on ibrutinib and likely harbor *BTK* mutations, as the binding site of ibrutinib and acalabrutinib is identical [107].

Despite the durable responses that can be achieved by optimally sequencing novel agents, combinations might be even more promising. Recently, concurrent *BTK*, *PLCG2* and *BCL2* mutations were found in patients who were treated with continuous ibrutinib monotherapy, had relapsed and then received continuous single-agent venetoclax [108]. Upon progression on venetoclax, four of eight evaluable patients with CLL-type progression harbored both BTKi-specific (*BTK/PLCG2*) and venetoclax-specific (*BCL2*) resistance mutations, suggesting that sequencing of single agents might lead to a situation in a substantial fraction of patients in which re-exposure to either of the drugs would probably be unsuccessful.

### 6.2. Other B-Cell Receptor Pathway-Targeting Approaches

PI3K inhibitors have been investigated as monotherapy (duvelisib) or in combination (idelalisib plus rituximab, umbralisib plus ublituximab) [50,51,109,110]. However, no specific information on their efficacy in patients progressing on ibrutinib and/or venetoclax has been published so far, as patients who were pretreated with BTKi or venetoclax were excluded from most of these studies.

In the setting of ibrutinib-resistant CLL mediated by *PLCG2* mutations, in vitro analyses have shown that inhibition of SYK and LYN, both upstream of BTK, can overcome persistent survival signaling [111]. In a recent phase 2 study, the SYK inhibitor entospletinib has produced responses in patients previously treated with B-cell receptor pathway inhibitors, even in patients harboring *BTK* and *PLCG2* mutations [112]. The overall response rate was however low (33%) and the progression-free survival was short. In the CLLRUmbrella2 study, a combination treatment of entospletinib and the BTK inhibitor tirabrutinib with or without the addition of obinutuzumab was evaluated in patients with relapsed/refractory CLL [113]. The overall response rates at week 25 were 100% for the double combination and 90% for the triple combination, undetectable MRD was only achieved by 10% of patients in the triple combination group [113]. Cerdulatinib, a SYK/JAK-STAT inhibitor has shown activity specifically in ibrutinib-resistant CLL samples [114]. In the first in human study, the drug showed promising activity against CLL, although not in patients that had progressed on ibrutinib before starting cerdulatinib treatment [115].

The inhibition of other proteins within the B-cell receptor pathway has also produced encouraging results. PKCβ inhibitors as well as MALT1 inhibitors have been shown to effectively kill ibrutinib-resistant CLL cells in vitro, though clinical data on these drugs have not been published yet [116,117].

### 6.3. Other Currently Investigated Non-Cellular Experimental Treatments

Based on the observations that venetoclax resistance appears to be mediated by an upregulation of the anti-apoptotic proteins Mcl-1 and to a lesser extent Bcl-X_L_ these proteins are considered as promising targets to overcome resistance to Bcl-2 inhibition [23,24,73,75]. AMG-176, a direct Mcl-1 antagonist has shown activity in a preclinical setting by effectively killing CLL cells while sparing normal blood cells and showing synergy with venetoclax; however, a phase 1 study of the drug had to be suspended due to safety concerns [118]. Another direct Mcl-1 inhibitor, AZD5991, is currently studied in a phase 1/2 trial after demonstrating potent antitumor activity in vitro and in preclinical models of acute myeloid leukemia (AML) and multiple myeloma [119]. CDK9 is the transcriptional regulator of Mcl-1 expression and the CDK9 inhibitor voruciclib has indeed shown to effectively reduce Mcl-1 expression in preclinical studies, a phase 1 study in patients with B-cell malignancies or AML is currently recruiting [120,121]. The above mentioned Bcl-X_L_ degrader might also be a promising approach to target upregulation of anti-apoptotic proteins often observed under venetoclax treatment [102].

Another therapeutic approach is targeted by cirmtuzumab, which is a humanized monoclonal antibody targeting Receptor Tyrosine Kinase Like Orphan Receptor 1 (ROR1). ROR1 is highly expressed on CLL cells but not on normal tissue and it acts as a receptor for Wnt5a which was found to enhance CLL cell proliferation [122,123]. The antibody has been evaluated in patients with relapsed/refractory CLL alone and in combination with ibrutinib [122,124,125]. While single-agent cirmtuzumab has not produced any objective responses, it led to decreasing lymphocyte counts in the majority of patients [122]. In combination with ibrutinib it showed an overall response rate of 67%. Whether this was solely attributable to the BTK inhibitor will be shown in a randomized phase 2 study of ibrutinib vs. ibrutinib plus cirmtuzumab that is currently ongoing.

Another promising target on the B-cell surface is the receptor of the B-cell activating factor (BAFF-R), that appears to be constantly expressed throughout treatment with ibrutinib in contrast to CD20 [126]. The anti-BAFF-R antibody VAY-736 was found to enhance antibody-dependent cellular toxicity and block BAFF-mediated survival signaling in preclinical models of CLL [126]. In a phase 1 trial, VAY-736 was added to ibrutinib in patients with BTKi-specific resistance mutations (mainly *BTK* C481S) or insufficient responses to ibrutinib [127]. Six of 15 (40%) patients, including patients with resistance mutations, achieved complete remissions and three patients achieved undetectable MRD, allowing for ibrutinib discontinuation [127].

Other drug candidates have yielded encouraging activity in preclinical studies but have not yet been tested in clinical trials. For instance, a bromodomain and extra-terminal (BET) protein inhibitor, GS-5829, has demonstrated preclinical activity in CLL [128]. It effectively induced apoptosis and reduced proliferation in primary CLL cells while also inhibiting growth of NLCs, suggesting activity against the CLL-supportive microenvironment. Another BET inhibitor, JQ1, was shown to increase venetoclax-induced apoptotic effects in vitro and exhibit anti-tumor activity in venetoclax-resistant CLL cell lines [129]. Preclinical activity was also shown for Histone Deacetylase 6 (HDAC6) inhibition in CLL cell lines and euTCL1 transgenic mouse models, leading to the development and clinical testing of the selective HDAC6 inhibitor ACY-1215 in relapsed/refractory lymphoid malignancies [130,131].

Bispecific antibodies that are able to simultaneously bind antigens on effector and malignant cells, have also shown antitumor activity in CLL. A novel bispecific antibody targeting CD3 and CD19 induced potent T-cell mediated killing of CLL cells in vitro and in a patient-derived xenograft mouse model, whereas the established anti-CD3/CD19 antibody blinatumomab failed to induce a response in the same mouse model [132]. The bispecific anti-CD3 and anti-CD20 antibody epcoritamab has been shown to be highly active in different lymphoma entities ex vivo and is currently being evaluated within a clinical phase I trial for CLL patients with multiple relapses, as well as other lymphoma entities [133].

## 7. Conclusions

A steadily increasing number of patients with CLL is treated with novel agents, particularly BTK inhibitors and venetoclax with or without anti-CD20 antibodies. These patients will eventually relapse/progress and many of them will harbor resistance-conferring mutations, mostly in the genes encoding the target proteins of these novel agents. New treatment options are emerging for patients with resistance mutations, most prominently and currently most promising third-generation BTK inhibitors that appear to be effective after progression on ibrutinib and in patients with the most common acquired *BTK* mutations. However, most of these new options have not been tested in phase 3 trials yet and patients who sequentially progress on BTKi and venetoclax still pose a major challenge and are currently mostly offered cellular therapies.

As *BTK/PLCG2* and *BCL2* mutations are frequently acquired rather late in the course of mostly single-agent treatment, time-limited combination approaches aiming at undetectable MRD might be an excellent way to avoid the development of resistance mutations. In the absence of resistance-conferring mutations, patients could be successfully retreated with the same regimen. Systematic genetic analyses of patients relapsing after time-limited combinations and results of retreatment studies will provide answers to the question if time-limited combination treatments are indeed superior or equal to continuous monotherapy. The development of new treatment approaches in order to overcome resistance to targeted agents will be one of the major challenges in CLL research in the chemotherapy-free era, which has already begun.

## Figures and Tables

**Table 2:** Selected currently ongoing studies assessing time-limited combination approaches.

Name/Identifier	Experimental Treatment Arm	Phase	TN vs. R/R
FLAIR 2013-001944-76	Venetoclax + ibrutinib	3	TN
GLOW NCT03462719	Venetoclax + ibrutinib	3	TN
CLL13/GAIA NCT02950051	Venetoclax + rituximab Venetoclax + obinutuzumab Venetoclax + ibrutinib + obinutuzumab	3	TN
CLL17 NCT04608318	Venetoclax + obinutuzumab Venetoclax + ibrutinib	3	TN
ACE-CL-311 NCT03836261	Acalabrutinib + venetoclax ± obinutuzumab	3	TN
PreVent-ACaLL NCT03868722	Venetoclax + acalabrutinib	2	TN
CLL2-BAAG NCT03787264	(Bendamustine) + acalabrutinib + venetoclax + obinutuzumab	2	R/R
CLL2-BZAG NCT04515238	(Bendamustine) + zanubrutinib + venetoclax + obinutuzumab	2	R/R
CLLRUmbrella1 NCT02968563	Tirabrutinib + idelalisib ± obinutuzumab	2	R/R
CLLRUmbrella2 NCT02983617	Tirabrutinib + entospletinib ± obinutuzumab	2	TN, R/R
COSMOS NCT02639910	Tafasitamab + idelalisib/venetoclax	2	R/R

TN: treatment-naïve, R/R: relapsed/refractory.
